# Evaluation of Tetracycline Resistance and Determination of the Tentative Microbiological Cutoff Values in Lactic Acid Bacterial Species

**DOI:** 10.3390/microorganisms9102128

**Published:** 2021-10-11

**Authors:** Qingqing Ma, Zhangming Pei, Zhifeng Fang, Hongchao Wang, Jinlin Zhu, Yuan-kun Lee, Hao Zhang, Jianxin Zhao, Wenwei Lu, Wei Chen

**Affiliations:** 1 State Key Laboratory of Food Science and Technology, Jiangnan University, Wuxi 214122, China; 6190112075@stu.jiangnan.edu.cn (Q.M.); 7190112086@stu.jiangnan.edu.cn (Z.P.); zhifengf@foxmail.com (Z.F.); hcwang@jiangnan.edu.cn (H.W.); wx_zjl@jiangnan.edu.cn (J.Z.); zhanghao61@jiangnan.edu.cn (H.Z.); zhaojianxin@jiangnan.edu.cn (J.Z.); chenwei66@jiangnan.edu.cn (W.C.); 2 School of Food Science and Technology, Jiangnan University, Wuxi 214122, China; 3 Department of Microbiology & Immunology, Yong Loo Lin School of Medicine, National University of Singapore, Singapore 117545, Singapore; micleeyk@nus.edu.sg; 4 International Joint Research Laboratory for Pharmabiotics & Antibiotic Resistance, Jiangnan University, Wuxi 214122, China; 5 (Yangzhou) Institute of Food Biotechnology, Jiangnan University, Yangzhou 225004, China; 6 National Engineering Research Center for Functional Food, Jiangnan University, Wuxi 214122, China

**Keywords:** lactic acid bacteria, tetracycline resistance, minimum inhibitory concentration, tetracycline resistance gene, microbiological cutoff value

## Abstract

Lactic acid bacteria (LAB) are widely used as probiotics in the food industry owing to their beneficial effects on human health. However, numerous antibiotic resistance genes have been found in LAB strains, especially tetracycline resistance genes. Notably, the potential transferability of these genes poses safety risks. To comprehensively evaluate tetracycline resistance in LAB, we determined the tetracycline susceptibility patterns of 478 LAB strains belonging to four genera and eight species. By comparing phenotypes with genotypes based on genome-wide annotations, five tetracycline resistance genes, tet(M), tet(W/N/W), tet(L), tet(S), and tet(45), were detected in LAB. Multiple LAB strains without tetracycline resistance genes were found to be resistant to tetracycline at the currently recommended cutoff values. Thus, based on the minimum inhibitory concentrations of tetracycline for these LAB strains, the species-specific microbiological cutoff values for *Lactobacillus (para)gasseri*, *Lactobacillus johnsonii*, and *Lactobacillus crispatus* to tetracycline were first developed using the Turnidge, Kronvall, and eyeball methods. The cutoff values for *Lactiplantibacillus plantarum* were re-established and could be used to better distinguish susceptible strains from strains with acquired resistance. Finally, we verified that these five genes play a role in tetracycline resistance and found that tet(M) and tet(W/N/W) are the most widely distributed tetracycline resistance genes in LAB.

## 1. Introduction

Tetracyclines are widely used antibiotics in human medicine and animal husbandry owing to their broad-spectrum antibacterial activity, low production cost, and lack of serious adverse reactions [1]. However, with the extensive and unreasonable use of tetracyclines, bacterial tetracycline resistance has become a serious concern [2], and the acquisition of tetracycline resistance genes has been identified as the main cause of bacterial tetracycline resistance [3]. Most tetracycline resistance genes are dependent of the bacteria. So, usually the most frequent genes for Gram-negative bacteria are tet(A) and tet(B), which are relatively highly distributed [4,5]. Most tetracycline resistance genes are linked to transmissible plasmids, transposons, and conjugative transposons, which can quickly spread among bacteria in humans, animals, and the environment [6,7]. Accordingly, these genes pose a great threat to human and animal health [8].

Lactic acid bacteria (LAB) have been consumed for thousands of years and are “generally recognized as safe” microorganisms [9,10]. Several species of LAB have been granted the qualified presumption of safety (QPS) status [11]. However, in recent years, owing to the improper use of antibiotics (overuse and misuse), many LAB have developed drug resistance [12]. Several studies have revealed that LAB isolated from food harbor a variety of antibiotic resistance genes (ARGs) [13,14], which are located on the mobile genetic elements and have a potential risk of horizontal gene transfer [15,16]. When these strains enter the human intestinal tract with food, they may transmit their resistance genes to the intestinal pathogenic bacteria and opportunistic pathogenic bacteria through the food chain and confer drug resistance to hosts, thereby threatening human health. Some studies have also confirmed that the tetracycline resistance gene in LAB can be transferred to other bacteria through horizontal gene transfer [17]. In 2002, the Food and Agriculture Organization and the World Health Organization proposed that probiotics used for food consumption should be used to evaluate the safety of antibiotic resistance in commercial applications [18]. The European Food Safety Agency (EFSA) document highlighted the need to determine whether there is no acquired or transferable resistance factor in a candidate probiotic or starter culture to declare it safe for human and animal consumption and to obtain QPS status [19]. Moreover, the document proposes that antibiotic susceptibility testing should be conducted according to international standards, such as those of the International Standard Organization (ISO) and Clinical and Laboratory Standards Institute [20].

Antimicrobial susceptibility testing is a traditional method of drug resistance testing. These testing methods include K-B disk diffusion, broth macrodilution, broth microdilution, agar dilution, and E-test [21,22]. These culture-based tests determine the growth of bacteria in the presence of antibiotics and evaluate bacterial resistance based on the drug resistance phenotype [23]. Microbiological cutoff values (MCOFFs) are usually used as the interpretation criteria to identify antibiotic resistance and to differentiate strains with acquired resistance from susceptible strains [24]. However, the cutoff values of some LAB species have not yet been determined. The EFSA guidelines classify these LAB species according to their fermentation type and determine the cutoff values based on their fermentation type [20].

The drug resistance of bacteria is usually determined by sequencing of coding genes. With the breakthrough of genome-wide sequencing technology, researchers have developed a sequence alignment method to identify antimicrobial resistance genes through sequence similarity [25]. By comparing the nucleic acid sequence or protein sequence of a strain with the sequence in the antimicrobial resistance database, the ARGs in the genome of LAB strains can be quickly identified and characterized to evaluate their drug resistance and risk of transfer [26]. Furthermore, the transformation of drug resistance evaluation from phenotype-based to genotype-based was promoted by such comparisons.

This study aimed to determine the tetracycline sensitivity of eight species of LAB from different geographical locations and different sources and to establish a new sensitive-resistance cutoff value at the species level to distinguish sensitive strains without resistance genes from strains that have acquired resistance, using the sensitivity analysis and genotype association results. The epidemiology and species distribution of tetracycline resistance genes in LAB were determined through genotype-phenotype association analysis.

## 2. Materials and Methods

### 2.1. Strains and Cultural Conditions

Details of the 478 strains belonging to *Lacticaseibacillus paracasei* (*n* = 116), *Lacticaseibacillus rhamnosus* (*n* = 68), *Limosilactobacillus reuteri* (*n* = 47), *Lactiplantibacillus plantarum* (*n* = 99), *Lactobacillus (para)gasseri* (*n* = 100), *Lactobacillus johnsonii* (*n* = 18), or *Lactobacillus crispatus* (*n* = 30) are presented in Table S1. All strains were identified at the species level based on 16S rRNA sequencing and were held in cryotubes with 15%–30% (*w/v*) glycerol and deposited in the Culture Collection of Food Microorganisms (CCFM) of Jiangnan University. All LAB strains were grown in de Man, Rogosa, and Sharpe (MRS) liquid medium at 37 °C (*L. plantarum* at 28 °C) for 16–24 h. Before susceptibility testing, all strains were propagated for three generations under the specified culture conditions, as mentioned above.

### 2.2. Antibiotic Susceptibility Testing

The minimum inhibitory concentration (MIC) was determined by the microdilution broth method using hand-made 96-well plates, and the specific operation followed the international standard method ISO 10932 (IDF 223:2010) [27]. Briefly, 100 μL of serial two-fold dilutions of tetracycline were distributed into each well of the 96-well plates. The bacterial suspensions were diluted until the optical density (OD) was between 0.16 and 0.2 at 625 nm (Shimadzu UV-1800 spectrophotometer, Kyoto, Japan), with a corresponding concentration of 3 × 10^8^ CFU/mL. The suspensions were diluted again 1000 times, and then 100 μL was added to each well of the 96-well plates. The 96-well plates were incubated under anaerobic conditions at 37 °C (*L. plantarum* incubated at 28 °C) for 48 h. Tetracycline was purchased from Sangon Biotech (Shanghai, China). The MIC for each LAB strain was defined as the lowest antibiotic concentration without visible growth. MICs were measured in triplicate. *Lacticaseibacillus paracasei* ATCC 334 and *L. plantarum* ATCC 14917 served as quality control strains. The interpretation criteria used to differentiate wild-type strains from non-wild-type strains were defined as the MCOFFs by EFSA [20,28].

### 2.3. Identification of Tetracycline Resistance Genes

The genome sequences of the tested strains were aligned with the Comprehensive Antibiotic Resistance Database (CARD, http://arpcard.Mcmaster.ca (accessed on 22 August 2019)) through the Resistance Gene Identifier (RGI, version 5.1.0) to identify all known ARGs [29]. A gene was recognized as a putative ARG if the identity value at the amino acid level was not lower than 70%. Putative ARGs related to phenotypic resistance were plotted as a heatmap using TBtools v1.09852 [30]. The results of protein sequence alignment were visualized using ESPript 3.0.

### 2.4. Statistical Analysis and Determination of Tentative Microbiological Cutoff Values (TMCOFFs)

The TMCOFFs were determined using two classical statistical methods, as described by Turnidge et al. [31] and Kronvall [32]. The MIC cumulative frequency distribution table was obtained by statistical analysis of the MIC values. The MIC frequency distribution data were imported into ECOFFinder_XL_2010_v2.0 and Automatic_NRI-MIC_Win_V01beta data tables, respectively, according to the corresponding method instructions. The cutoff values containing 99% wild-type strains were rounded off to the adjacent twofold dilution antibiotic concentration and were defined as TMCOFFs in ECOFFinder Excel sheets. A visual method was also employed to formulate TMCOFFs. The visual method defined TMCOFFs as the MIC at the second twofold dilution concentration higher than the model MIC and that contained at least 95% of wild-type strains [33]. The final TMCOFFs obtained in this study were the median values of the cutoff values obtained from the three methods.

### 2.5. Sample Collection and RT-PCR

Lactic acid bacteria strains containing tetracycline resistance genes were inoculated into MRS liquid medium and incubated at 37 °C (*L. plantarum* incubated at 28 °C) to achieve logarithmic growth phase. Tetracycline was added at a final concentration of 1/2 × MIC (Table S1) of tetracycline. The bacterial culture without tetracycline was used as the control, and three independent biological repeats were employed in each group. The bacterial cells was collected by centrifugation at 2500× *g* for 10 min at 4 °C (Eppendorf 5424R centrifuge, Hamburg, Germany) after continuous culture for 1 h, and three biologically repetitive bacterial cells were mixed for subsequent RNA extraction [34].

Total RNA was extracted using the Bacteria RNA Extraction Kit (R403-01, Vazyme Biotech Co., Ltd., Nanjing, China) according to the manufacturer’s protocol. Three independent biological replicates were employed for each treatment, and the extracted RNA samples were mixed for each treatment. RNA purity and concentration were determined using an ultramicrospectrophotometer (Implen, Munich, Germany). RNA integrity was detected through agarose gel electrophoresis. Total RNA extracted from the bacteria was reverse transcribed into cDNA using HiScript III-RT SuperMix for qPCR (R323-01, Vazyme Biotech Co., Ltd., Nanjing, China) according to the manufacturer’s instructions.

By using the CFX connect real-time qPCR system (Bio-Rad, Hercules, CA, USA), the 16S rRNA gene as an internal reference gene, and the relative quantitative method, expression of the drug resistance gene was determined. All amino acid sequences of the same tetracycline resistance gene detected in this study were compared using the MEGA X v10.2.4 software to find the conserved sites of the sequence. The Primer-BLAST program (http://www.ncbi.nlm.nih.gov/tools/Primer-Blast/) (accessed on 10 October 2020) was used to design primers in the conserved region and check the specificity of primers [35]. The primers were synthesized by Shanghai Sunni Biotechnology Co., Ltd. The primer sequences were as follows: tet(M)-F, TTACTGTATCACCCGCTTCC; tet(M)-R, CAGTCGTCACATTCCAACC [36]; tet(W/N/W)-F, TGGAAAGACGACCTTGACGG; tet (W/N/W)-R, ACATCTGTGCCACTGGAAGG; tet(L)-F, CATTTGGTCTTATTGGATCG; tet (L)-R, ATTACACTTCCGATTTCGG; tet(S)-F, ACGCTATGGGTGTGAACAAGG; tet (S)-R, CAATAGGCGCAAGCATTCGG; 16S rRNA-F, AGAGTTTGATCCTGGCTCAG; 16S rRNA-R, CTACGGCTACCTTGTTACGA [37]; tet(45)-F, ACCTGCGAGTACAAACTGGG; and tet(45)-R, AACCCAATTACCGACCCGAA. The final reaction volume for RT-PCR was 10 μL, and the mixture comprised the following: 2 × iTaq^TM^ Universal SYBR^®^ Green Supermix (Bio-Rad, Hercules, CA, USA), 5 μL; forward primers (500 nM), 0.5 μL; reverse primers (500 nM), 0.5 μL; cDNA (100 ng), 1 μL; and ddH_2_O, 3 μL. The reaction conditions were 95 °C for 30 s; 95 °C, 5 s; 60 °C, 30 s; and 72 °C, 20 s. In the subsequent three steps, 39 cycles were performed, and the fluorescence was measured at 60 °C. The dissociation curve was generated under the following conditions: 65 °C, 5 s; 95 °C, 0.5 °C. Three biological replicates were set for each sample, and the experiment was repeated three times. A heatmap of the gene expression data was plotted using TBtools v1.09852 [38], and the expression level was log_2_ transformed [39].

## 3. Results

### 3.1. Determination of the MICs and Identification of the Resistance Phenotype

**Figure 1:**
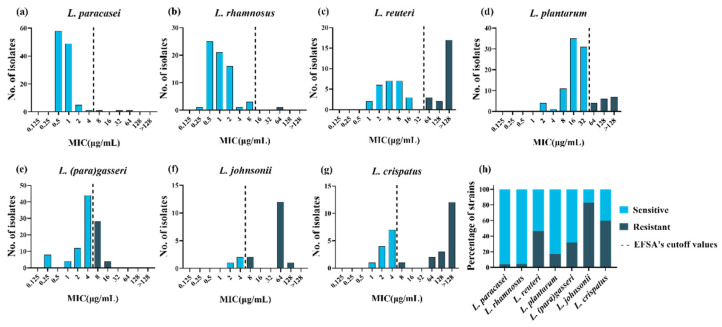
Minimum inhibitory concentration (MIC) distributions and drug resistance rate within 478 lactic acid bacteria strains. (**a**–**g**): Distribution of the MICs of tetracycline for eight lactic acid bacterial species. The black dotted lines represent epidemiological cutoff values reported by the European Food Safety Agency (EFSA). Sky blue represents phenotypically sensitive strains, and blue-black represents phenotypically resistant strains. (**h**) Distribution of tetracycline-resistant (black) and -susceptible (gray) strains according to the EFSA epidemiological cutoff values.

To explore the tolerance level of different species of LAB to tetracycline, the MICs of tetracycline were tested using the broth microdilution method for 478 LAB strains. The MICs of tetracycline for the two quality control strains in this study were within the quality control range, and the MICs for all strains are presented in Table S1. Differences were observed in the intra- and interspecies levels of tetracycline susceptibility. *Lactiplantibacillus plantarum* and *L. reuteri* had a wide MIC range that covered 8 twofold dilutions compared to the remaining species. Furthermore, *L. paracasei*, *L. rhamnosus* and *L. crispatus* covered 7 twofold dilutions, while *L. johnsonii* and *L.* *(para)gasseri* covered only 5–6 twofold dilutions (Figure 1a–g). The MICs for *L. paracasei* and *L. rhamnosus* were mainly between 0.5 and 2 μg/mL, while those for *L. reuteri*, *L.* *(para)gasseri*, *L. johnsonii,* and *L. crispatus* were mainly between 2 and 8 μg/mL. In particular, compared to other species, *L. plantarum* showed higher MICs, ranging from 8 to 32 μg/mL. The MICs for *L. johnsonii*, *L. crispatus,* and *L. reuteri* showed an obvious bimodal distribution, suggesting that these species may contain acquired tetracycline resistance genes.

Phenotypic resistance was interpreted based on the MCOFFs reported by EFSA, which served as the interpretation criteria to distinguish susceptible strains free of phenotypically discoverable acquired resistance mechanisms from resistant strains. Generally, a strain was classified as susceptible when the MIC of a given antibiotic was not more than the established MCOFF. Herein, 23% (108/478) of the LAB strains were resistant to tetracycline; most *L. johnsonii* and *L. crispatus* strains showed tetracycline resistance, with resistance levels of 83% (15/18) and 60% (18/30), respectively. *Lacticaseibacillus rhamnosus* and *L. paracasei* showed the least common phenotypic resistance, with tetracycline resistance observed in 1% (1/68) and 3% (3/116) of the strains examined, respectively. *Lactiplantibacillus* *plantarum*, *L.* *(para)gasseri,* and *L. reuteri* exhibited tetracycline resistance levels of 17% (17/99), 32% (32/100), and 47% (22/47), respectively (Figure 1h).

### 3.2. Identification of ARGs and Their Correlation with Phenotype

The MCOFF is often used as an interpretation standard for the separation of sensitive strains from acquired resistance strains. If the MIC of one or more antibiotics for one strain is greater than the cutoff value, it is necessary to further explore its resistance mechanism at the genetic level [18]. Therefore, the genome sequences of 478 strains of LAB, which were tested for phenotypic resistance, were subjected to sequence alignment with CARD. Based on the selection standards, among the 478 strains, five tetracycline resistance-related genes encoded tetracycline target protection proteins (tet(W/N/W), tet(M), and tet(S)) and efflux pumps (tet(45) and tet(L)).

**Figure 2:**
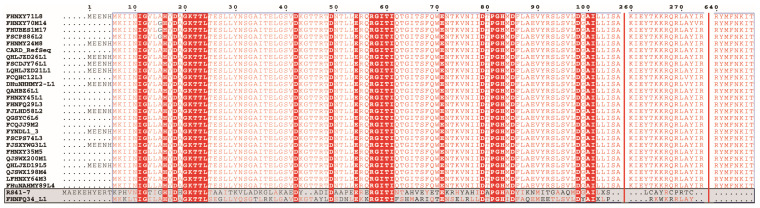
Multi-sequence alignment of tet(M) sequences of *Lactiplantibacillus plantarum* RS41-7 and *Lactobacillus gasseri* FHNFQ34_L1 (the bottom two sequences) with the tet(M) sequences of other strains in this study.

**Figure 3:**
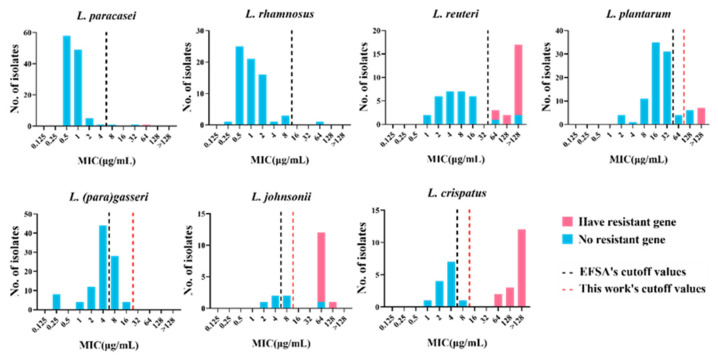
MIC distribution of 478 lactic acid bacteria strains with or without the tetracycline resistance gene. Blue: strains without resistance gene. Pink: strains with the tetracycline resistance gene. Black dotted line: cutoff value established by EFSA. Red dotted line: the new cutoff value established by this work.

To identify the resistance determinants of tetracycline-resistant strains, we analyzed the association between phenotypically resistant strains and their genotypes (Table 1). Among the three tetracycline-resistant strains of *L. paracasei*, the strain FCQHC12L3 was characterized by the presence of tet(M). However, no tetracycline resistance gene was found in the remaining two phenotypically resistant strains. Among the 17 tetracycline-resistant strains of *L. plantarum*, six were characterized by the presence of tet(M), while QHLJZD13-L6 was characterized by the presence of tet(S), and no related resistance genes were detected in 10 strains. Among the 22 tetracycline-resistant strains of *L. reuteri*, two strains were characterized by the presence of tet (M) and tet(L), whereas two strains were characterized by the presence of tet (W/N/W) and tet(L). Three strains and eleven strains harbored only tet(M) or tet(W/N/W), respectively. Furthermore, strain FYNLJ83L8 was characterized by the presence of tet(45), and three strains were not associated with resistance genes. Across the 15 tetracycline-resistant strains of *L. johnsonii*, the gene tet(W/N/W) was found in 11 resistant strains. In addition, FHNXY70M2 was characterized by the presence of tet(W/N/W) and tet (L), and the remaining three strains did not display resistance-related genes. Of the 18 resistant strains of *L. crispatus*, tet (M) was found in two resistant strains, and tet(W/N/W) was found in four resistant strains. Ten strains were characterized by the presence of tet(W/N/W) and tet(L), and the strain FHNXY70M14 was characterized by the presence of tet(M), tet(W/N/W), and tet(L). However, no resistance-related genes were detected in strain FHNXY56M7. No tetracycline resistance genes were found in any of the 32 tetracycline-resistant strains of *L.* *(para)gasseri* and one tetracycline-resistant strain of *L. rhamnosus*. In particular, two strains (*L. plantarum* RS41-7 and *L. gasseri* FHNFQ34_L1) harbored a tet(M) gene but were sensitive to tetracycline. Furthermore, the tet(M) sequence of the two strains had obvious deletions in the functional sites, resulting in the loss of resistance function (Figure 2). Therefore, in this study, we did not classify these two strains as carriers of the gene tet(M). In brief, through genotypic and phenotypic association analysis, these five tetracycline resistance genes could explain the resistance phenotypes of 33% (1/3) of *L. paracasei*, 41% (7/17) of *L. plantarum*, 80% (12/15) of *L. johnsonii*, 94% (17/18) of *L. crispatus*, and 86% (19/22) of *L. reuteri* strains. However, resistance phenotypes of 48% (52/108) of the resistant strains could not be explained based on their genotypes (Figure 3).

### 3.3. Definition of New Susceptibility–Resistance Cutoff Values

The results of genotype-phenotype association analysis revealed that the genetic basis for the resistance of 48% (52/108) of the strains with tetracycline resistance phenotype could not be determined, including that of two strains of *L. paracasei*, one strain of *L. rhamnosus*, 10 strains of *L. plantarum*, one strain of *L. crispatus*, three strains of *L. reuteri*, three strains of *L. johnsonii,* and 32 strains of *L.* *(para)gasseri. Lactobacillus* *(para)gasseri* has a high drug resistance rate; however, the resistance determinants of all phenotypically resistant strains could not be identified. We speculate that the cutoff value based on the fermentation type may not be applicable to all species of LAB. Thus, it is recommended to develop MCOFFs at the species level. *Lactobacillus* *(para)gasseri*, *L. crispatus*, and *L. johnsonii* did not have species-specific cutoff values; therefore, we statistically analyzed the MIC frequency distribution of these species of *Lactobacillus* to establish species-specific TMCOFFs, which could better distinguish the resistant strains from the sensitive strains without acquired ARGs. *Lacticaseibacillus paracasei*, *L. rhamnosus*, *L. plantarum,* and *L. reuteri* had MCOFFs at the species level; however, 10% of *L. plantarum* had resistance phenotype but no resistance determinants. Therefore, we reformulated the cutoff value of *L. plantarum* to determine whether the strains containing resistance genes could be better distinguished from sensitive strains. Based on the MIC distribution of tetracycline of *L*. *(para)gasseri*, *L. crispatus*, *L. johnsonii,* and *L. plantarum* in this study, two different statistical approaches (Turnidge and Kronvall) and a “visual estimation” approach (eyeball method) were used to determine the new susceptibility–resistance cutoff values (Table 2).

Based on the new cutoff values, all *L.* *(para)gasseri* strains were classified as sensitive, and the strains of *L. crispatus* containing resistance genes were distinguished from the sensitive strains without acquired ARGs. One strain belonging to *L. johnsonii* was not associated with the tetracycline resistance gene and was classified as phenotypically resistant. However, the MIC for this strain was found to be equivalent to that for another *L. johnsonii* strain containing the tetracycline resistance gene. Accordingly, we speculated that it contained potential tetracycline resistance genes. Therefore, the new cutoff value could completely distinguish the strains containing resistance genes from sensitive strains in *L. johnsonii*. The cutoff value of tetracycline for *L. plantarum* was newly formulated as 64 μg/mL, which is the same as that formulated by Flórez et al. [40]. However, the resistance determinants of six strains of *L. plantarum* still need to be further explored.

### 3.4. Prevalence and Distribution of Tetracycline Resistance Genes in LAB

To explore the distribution and prevalence of these five tetracycline resistance genes in LAB, we performed statistical analysis on the detection of tetracycline resistance genes in eight species of LAB (Table 3). The most widely distributed tetracycline resistance gene in LAB was tet(M), which was detected in four LAB species, including *L. paracasei*, *L. plantarum*, *L. reuteri*, and *L. crispatus*. The genes tet(W/N/W) and tet(L) were detected in three species of LAB, including *L. reuteri*, *L. johnsonii,* and *L. crispatus*. The tet(S) gene was detected in only one strain of *L. plantarum,* and the tet (45) gene was found in only one strain of *L. reuteri*. The tet(W/N/W) gene was the most frequently detected tetracycline resistance gene in LAB; it was detected in 30 strains of LAB in this study. The genes tet(M), tet(L), tet(S), and tet(45) had detection frequencies of 26, 16, 1, and 1, respectively. Most types of the tetracycline resistance genes were detected in *L. reuteri*, including tet(M), tet(W/N/W), tet(L), and tet(45). Three of the tetracycline resistance genes were detected in *L. crispatus*, namely tet(M), tet(W/N/W), and tet(L). Two genes, tet(W/N/W) and tet(L), were detected in *L. johnsonii*. Two tetracycline resistance genes, tet(M) and tet(S), were found in *L. plantarum*. Among *L. paracasei* strains, only one harbored the tet(M) gene. No tetracycline resistance genes were found in any of the *L. rhamnosus* and *L. (para)gasseri* strains in this study. Herein, the tetracycline resistance gene was detected in 12% (56/478) of the strains. A total of 26 LAB strains contained only the tet(W/N/W) gene, while 12 LAB strains contained only the tet(M) gene. Interestingly, the gene tet(L) did not appear alone in LAB but was always detected together with other tetracycline resistance genes. The genes tet(M) and tet(L) were detected together in 12 strains of LAB. The genes tet(W/N/W) and tet(L) were detected concurrently in three strains of LAB. Three tetracycline resistance genes, tet(M), tet(W/N/W), and tet(L), were detected simultaneously in one *L. crispatus* strain. One strain of *L. plantarum* contained only the tet(S) gene, and one *L. reuteri* strain contained only the tet(45) gene.

The genes of tet(M) and tet(W/N/W) are the most widely distributed tetracycline resistance genes in LAB. In order to further explore their phylogenetic relationship, multiple sequence alignments of a total of 25 tet(M) and 30 tet(W/N/W) gene sequences identified in this paper together with the same genotype sequences retrieved from the NCBI database were performed by ClustalW, and the phylogenetic tree was constructed using the Neighbor-Joining method by MEGA X (Figures S1 and S2). Most of the same species were in the same branch, suggesting that the genes had adaptive mutations when transferred to different species, and the tet(M) and tet(W/N/W) genes of LAB of the same species may have come from the same host.

### 3.5. Expression of Tetracycline Resistance Gene Based on RT-PCR

**Figure 4:**
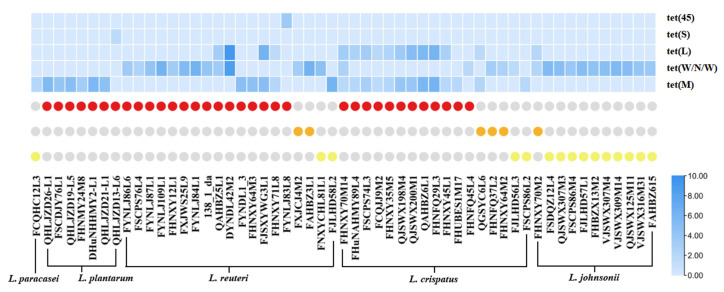
The expression of resistance genes in phenotypically resistant strains induced by tetracycline at the concentration of 1/2 × MIC. The heatmap above represents the level of gene expression. The circle chart represents the MIC for the strain, the red circle indicates that the MIC for the corresponding strain is greater than 128 μg/mL, the orange circle indicates that the MIC for the corresponding strain is equal to 128 μg/mL, and the yellow circle indicates that the MIC for the corresponding strain is equal to 64 μg/mL.

To verify that the five tetracycline resistance genes play a role in the resistance to tetracycline, the expression of drug resistance genes carried by drug-resistant strains induced by tetracycline at a concentration of 1/2 × MIC was determined using RT-PCR. After being induced by tetracycline, the expression of these five drug-resistant genes in the drug-resistant strains was upregulated, indicating that these genes are indeed involved in the resistance to tetracycline. In the same species of LAB, some strains with the same resistance determinants had different MICs; this result may be attributed to the differences in gene expression. However, assessing the relative expression of genes alone cannot completely explain this phenomenon. We also attempted to directly compare the expression of the same resistance genes in different strains; however, the strains with the same resistance determinants had different MICs, which could not be explained; we thus speculated that it may be mainly related to gene structure [41]. Interestingly, the expression level of the tet (L) gene was similar to that of other tetracycline resistance genes in the same strain, suggesting that these genes may be located in the same gene cluster (Figure 4).

## 4. Discussion

At present, there are many LAB species with QPS status for which MCOFFs at the species level have not been determined. However, many researchers have found that different LAB species have different tolerances to the same antibiotic [42]. Species-specific cutoff values must be set when determining phenotypic resistance. Studies have shown, based on the cutoff values recommended by the EFSA at the species level, that some LAB species have high resistance levels [43], suggesting that these cutoff values should be reexamined according to the genetic basis for resistance. Therefore, we formulated a species-specific cutoff value of tetracycline for *L.* *(para)gasseri*, *L. crispatus,* and *L. johnsonii*. The drug resistance rate of *L. plantarum* was relatively high. Furthermore, the determinants of tetracycline resistance could not be found for several phenotypically resistant strains. The cutoff values formulated by EFSA for *L. plantarum* have only been defined for strains used in animal feed [44]. The strains used in the present study were of different origins. Based on the MIC distribution data for the abovementioned four species, we developed a new cutoff value. Similar results for both the absolute MIC and MIC ranges for these species have been reported by other authors [45].

The newly established cutoff value for *L. plantarum* is greater than that established by EFSA but equivalent to that formulated by Flórez et al. [40]. The newly established cutoff values for *L.* *(para)gasseri*, *L. crispatus,* and *L. johnsonii* were also greater than those established by EFSA according to the fermentation metabolism category. The new cutoff values can completely distinguish the sensitive strains of *L.* *(para)gasseri*, *L. crispatus, L. johnsonii*, and *L. plantarum* from the strains with acquired resistance genes, except one strain of *L. johnsonii* (MIC = 64 μg/mL) and six strains of *L. plantarum* (MIC = 128 μg/mL), which were suspected to possess acquired resistance to this antibiotic; we are currently exploring their resistance using the transcriptional technology [46]. Among the remaining three species of LAB, six phenotypically resistant strains that were not associated with known tetracycline resistance genes were present. These LAB strains may carry other genes that have not been found to play a role in the function of tetracycline resistance before. We also used transcriptome analysis to explore whether their resistance phenotypes are mediated by unknown tetracycline resistance genes, evolution, or other internal factors [47], which made it difficult to predict the resistance phenotype by relying on the database, to determine whether the existing cutoff value is reasonable.

To explore the resistance determinants of phenotypically resistant strains, we compared the protein sequences of all strains with the CARD. Sequences with similarity greater than 30% are believed to be homologous; however, when an identity >30% was used as the threshold to screen resistance genes, all phenotypically sensitive strains had multiple tetracycline resistance genes (Figure S3). Thus, we gradually increased the threshold with a gradient of 10% and found that when identity was > 70%, all phenotypically sensitive strains did not have tetracycline resistance genes. As a result, the threshold was set at 70%. Among the phenotypically resistant strains with tetracycline resistance genes, five key genes, tet(M), tet(W/N/W), tet(L), tet(S), and tet(45), related to tetracycline resistance phenotype were identified in their genomes. In particular, among all LAB strains in this study, the genes tet(M) and tet(W/N/W) were the most widely distributed tetracycline resistance genes [48] and were usually located on mobile genetic elements. The genes tet(Q), tet(K), and tet(O) were reported to be detected in LAB strains [49,50]; however, they were not detected in this study; this result may be related to the species, region, and source of LAB [13]. The common mechanisms of tetracycline resistance include ribosome protection, antibiotic efflux, and antibiotic inactivation [51]. Among all tetracycline-resistant strains in this study, the resistance mechanism of encoding ribosomal protective protein was dominant, followed by the efflux pump protein, indicating that tetracycline resistance is mainly mediated by the acquisition of tetracycline resistance genes encoded by the two resistance mechanisms, and that the two mechanisms work together to enhance the resistance of bacteria.

## 5. Conclusions

Based on the MIC distribution data of 478 strains of LAB, LAB showed moderate resistance to tetracycline. Further, formulating the breakpoint value at the species level was found to be necessary. Therefore, the species-specific microbiological cutoff values for *L. (para)gasseri*, *L. crispatus*, and *L. johnsonii* against tetracycline were formulated, and new susceptibility-resistance cutoff values for *L. plantarum* were defined. The genes tet(M), tet(W/N/W), tet(L), tet(S), and tet(45) were the key resistance genes for the tetracycline resistance phenotype and were found to widely exist in LAB. The determination of antibiotic resistance in probiotic strains is related to food safety issues. The findings of this study provide certain guiding significance and reference values at the phenotype and genotype levels for the safe application of LAB in the food industry and the formulation of probiotic resistance evaluation standards.

## Figures and Tables

**Table 1:** Association between tetracycline-resistant strains and tetracycline resistance genes in eight lactic acid bacterial species.

Tetracycline Resistance Genes	Number of Tetracycline-Resistant Strains
tet(M)	*L. paracasei* (1), *L. plantarum* (6), *L. reuteri* (3), *L.crispatus* (2)
tet(W/N/W)	*L. reuteri* (11), *L.johnsonii* (11), *L.crispatus* (4)
tet(S)	*L. plantarum* (1)
tet(45)	*L. reuteri* (1)
tet(M) and tet(L)	*L. reuteri* (2), *L.crispatus* (10)
tet(W/N/W) and tet(L)	*L. reuteri* (2), *L.johnsonii* (1)
tet(M), tet(W/N/W), and tet(L)	*L.crispatus* (1)
No tetracycline resistance genes	*L. paracasei* (2), *L. rhamnosus* (1), *L. plantarum* (10), *L. reuteri* (3), *L.johnsonii* (3), *L.crispatus* (1), *L. (para)gasseri* (32)

Number in parentheses represents the number of strains with tetracycline resistance genes detected in the corresponding lactic acid bacterial species.

**Table 2:** Comparison of tentative microbiological cutoff values (TMCOFFs) for tetracycline calculated using two statistical methods and the eyeball method.

		TMCOFFs Obtained Using the Indicated Method (%) ^a^			
Species	EFSA Cut Off	Method of Turnidge et al. ^b^	Method of Kronvall	Eyeball Method	Median for the Method
*L. (para)gasseri*	4 (68%)	16 (100%)	256 (100%)	16 (100%)	16 (100%)
*L. johnsonii*	4 (17%)	32 (38%)	16 (38%)	16 (38%)	16 (38%)
*L. crispatus*	4 (40%)	8 (50%)	16 (50%)	16 (50%)	16 (50%)
*L. plantarum*	32 (83%)	64 (87%)	64 (87%)	64 (87%)	64 (87%)

^a^ Values in percentages denote the proportion of isolates with an MIC that is not greater than the TMCOFFs. ^b^ Calculated TMCOFFs including 99% of the strains in the wild-type population.

**Table 3:** The detailed distribution of the detected tetracycline resistance genes in different lactic acid bacterial species.

Species	Total Strain Number	TET^R^	tet(M)	tet(W/N/W)	tet(L)	tet(S)	tet(45)
*L. paracasei*	116	3	1	0	0	0	0
*L. rhamnosus*	68	1	0	0	0	0	0
*L. plantarum*	99	17	6	0	0	1	0
*L. reuteri*	47	22	5	13	4	0	1
*L. johnsonii*	18	15	0	12	1	0	0
*L. crispatus*	30	18	13	5	11	0	0
*L.* *(para)gasseri*	100	32	0	0	0	0	0
Total	478	108	25	30	16	1	1

TET^R^ represents the number of tetracycline-resistant strains based on the EFSA breakpoint value.

## Data Availability

Of the 478 LAB strains deposited in the NCBI GenBank database, 439 were released as part of our previous studies [52,53,54,55], and the remaining 39 genomes were deposited under project accession no. PRJNA658852. The accession numbers for all individual genomes used in this study are presented in Table S1.

## Supplementary Materials

The following are available online at https://www.mdpi.com/article/10.3390/microorganisms9102128/s1, Table S1. Details of 478 strains belonging to 8 lactic acid bacterial species, including their origin, MIC values, resistance phenotype, and accession number. Figure S1. Phylogenetic analysis of tet(M) belonging to 25 strains of lactic acid bacteria identified in this paper together with the same genotype sequences retrieved from NCBI database. Figure S2. Phylogenetic analysis of tet(W/N/W) belonging to 30 strains of lactic acid bacteria identified in this paper together with the same genotype sequences retrieved from NCBI database. Figure S3. The resistance genes of 478 strains of lactic acid bacteria were detected with an identity greater than 30% as the threshold.
