# Supplementary material for: Evaluation of Tetracycline Resistance and Determination of the Tentative Microbiological Cutoff Values in Lactic Acid Bacterial Species
Source: Microorganisms. 2021 Oct 11;9(10):2128. doi: 10.3390/microorganisms9102128 (PMC8538481; doi:10.3390/microorganisms9102128)
Supplement: Supplementary file 1 [file microorganisms-09-02128-s001.zip › microorganisms-1405495-supplementary.pdf]

Supplementary data

Table S1. Details of 478 strains belonging to 8 lactic acid bacterial species, including their origin, minimum inhibitory concentration (MIC) values, resistance phenotype, and accession number.

Species	Strain ID	Cutoff value	MIC (μg/mL)	Phenotype	Source	Region	Accession number
L. paracasei	ATCC 334	1 to 4	2	S	-	-	-
L. plantarum	ATCC 14917	8 to 32	16	S	-	-	-
L. paracasei	FCQHC12L3	4	64	R	Human feces	Chongqing	SAMN15891445
L. paracasei	FQHXN23L6	4	32	R	Human feces	Qinghai	SAMN15891481
L. paracasei	VCQQJ30173L8	4	8	R	Chinese pickle	Chongqing	SAMN15891541
L. paracasei	VCQYB7-171M7	4	4	S	Chinese pickle	Chongqing	SAMN15891544
L. paracasei	FHeNXX1L1	4	2	S	Human feces	Henan	SAMN15891462
L. paracasei	FZJHZ11L2	4	2	S	Human feces	Zhejiang	SAMN15891510
L. paracasei	V_CQWX3_102_L7	4	2	S	Chinese pickle	Chongqing	SAMN15891533
L. paracasei	V_CQWX5_L7	4	2	S	Chinese pickle	Chongqing	SAMN15891535
L. paracasei	FNMHLBE25M1	4	2	S	Human feces	Inner Mongolia	SAMN15891480
L. paracasei	17005	4	1	S	Chinese pickle	Chongqing	SAMN15891425
L. paracasei	FFJND1L2	4	1	S	Human feces	Fujian	SAMN15891456
L. paracasei	FFJFZ2L5	4	1	S	Human feces	Fujian	SAMN15891471
L. paracasei	DP11	4	1	S	Chinese pickle	Sichuan	SAMN15891429
L. paracasei	FFJNDD7M6	4	1	S	Human feces	Fujian	SAMN15891430
L. paracasei	FZJHZ2D2M1	4	1	S	Human feces	Zhejiang	SAMN15891431
L. paracasei	FBJSY11L4	4	1	S	Human feces	Beijing	SAMN15891440
L. paracasei	FBJSY22L1	4	1	S	Human feces	Beijing	SAMN15891441
L. paracasei	FFJLY55L1	4	1	S	Human feces	Fujian	SAMN15891455
L. paracasei	FFJND15L2	4	1	S	Human feces	Fujian	SAMN15891457
L. paracasei	FGDLZ41	4	1	S	Human feces	Guangdong	SAMN15891460
L. paracasei	FHNFQ4L3	4	1	S	Human feces	Henan	SAMN15891463
L. paracasei	FHNMY65L3	4	1	S	Human feces	Henan	SAMN15891470
L. paracasei	FJSSZ4L8	4	1	S	Human feces	Jiangsu	SAMN15891474
L. paracasei	FQHXN78L2	4	1	S	Human feces	Qinghai	SAMN15891482
L. paracasei	FQHXN84L6	4	1	S	Human feces	Qinghai	SAMN15891485
L. paracasei	FQHXN89L3	4	1	S	Human feces	Qinghai	SAMN15891487
L. paracasei	FQHXN96L1	4	1	S	Human feces	Qinghai	SAMN15891489
L. paracasei	FSDLZ14M1	4	1	S	Human feces	Shandong	SAMN15891496
L. paracasei	FSDLZ35M1	4	1	S	Human feces	Shandong	SAMN15891499
L. paracasei	FSDWH1L4	4	1	S	Human feces	Shandong	SAMN15891502
L. paracasei	FSDYT1-4	4	1	S	Human feces	Shandong	SAMN15891503
L. paracasei	FZJHZ22L2	4	1	S	Human feces	Zhejiang	SAMN15891511
L. paracasei	FZJHZD11L5	4	1	S	Human feces	Zhejiang	SAMN15891514

L. paracasei	FZJHZD29L1	4	1	S	Human feces	Zhejiang	SAMN15891515
L. paracasei	FJSWX3L3	4	1	S	Human feces	Jiangsu	SAMN15891520
L. paracasei	RS241	4	1	S	Chinese pickle	Sichuan	SAMN15891532
L. paracasei	RS8-5	4	1	S	Chinese pickle	Sichuan	SAMN15891529
L. paracasei	VCQBB4-126L6	4	1	S	Chinese pickle	Chongqing	SAMN15891537
L. paracasei	VCQJLP2-131L2	4	1	S	Chinese pickle	Chongqing	SAMN15891539
L. paracasei	FQHXN98L4	4	1	S	Human feces	Qinghai	SAMN15891490
L. paracasei	FQHXN113L1	4	1	S	Human feces	Qinghai	SAMN15891492
L. paracasei	FCQHC25L1	4	1	S	Human feces	Chongqing	SAMN15891446
L. paracasei	FCQNA15L3	4	1	S	Human feces	Chongqing	SAMN15891448
L. paracasei	FCQNA33L2	4	1	S	Human feces	Chongqing	SAMN15891451
L. paracasei	FCQNA42L2	4	1	S	Human feces	Chongqing	SAMN15891454
L. paracasei	FNMHLBE12L1	4	1	S	Human feces	Inner Mongolia	SAMN15891479
L. paracasei	FCQNA30M5	4	1	S	Human feces	Chongqing	SAMN15891450
L. paracasei	FXJWS7M1	4	1	S	Human feces	Xinjiang	SAMN15891507
L. paracasei	FXJWS3M2	4	1	S	Human feces	Xinjiang	SAMN15891506
L. paracasei	FZJHZD7L8	4	1	S	Human feces	Zhejiang	SAMN15891513
L. paracasei	FXJWS28L1	4	1	S	Human feces	Xinjiang	SAMN15891508
L. paracasei	FZJHZ2M2	4	1	S	Human feces	Zhejiang	SAMN15891509
L. paracasei	FCQNA35M4	4	1	S	Human feces	Chongqing	SAMN15891452
L. paracasei	VCQQJ4-174M3	4	1	S	Chinese pickle	Chongqing	SAMN15891540
L. paracasei	VCQY0Y1-157M2	4	1	S	Chinese pickle	Chongqing	SAMN15891543
L. paracasei	VCQRC7-161M2	4	1	S	Chinese pickle	Chongqing	SAMN15891542
L. paracasei	VCQBB3-125L12	4	1	S	Chinese pickle	Chongqing	SAMN15891536
L. paracasei	VCQWX4-103L1	4	1	S	Chinese pickle	Chongqing	SAMN15891534
L. paracasei	104S2	4	0.5	S	Chinese pickle	-	SAMN15891424
L. paracasei	FZJZJ3L4	4	0.5	S	Human feces	Zhejiang	SAMN15891518
L. paracasei	FZJJH2L6	4	0.5	S	Human feces	Zhejiang	SAMN15891516
L. paracasei	FZJJH3L1	4	0.5	S	Human feces	Zhejiang	SAMN15891517
L. paracasei	FHNMY13M7	4	0.5	S	Human feces	Henan	SAMN15891465
L. paracasei	FHNMY20M6	4	0.5	S	Human feces	Henan	SAMN15891466
L. paracasei	FHNMY59L1	4	0.5	S	Human feces	Henan	SAMN15891467
L. paracasei	FHNMY60L3	4	0.5	S	Human feces	Henan	SAMN15891468
L. paracasei	FHNMY61L4	4	0.5	S	Human feces	Henan	SAMN15891469
L. paracasei	FAHBZ47M5	4	0.5	S	Human feces	Anhui	SAMN15891432
L. paracasei	FAHBZ60M3	4	0.5	S	Human feces	Anhui	SAMN15891433
L. paracasei	FGDLZ5M2	4	0.5	S	Human feces	Guangdong	SAMN15891458
L. paracasei	FGDLZ6M8	4	0.5	S	Human feces	Guangdong	SAMN15891459
L. paracasei	DJXSRYG2L10	4	0.5	S	Chinese	Jiangxi	SAMN15891428

					fermented sauce		
L. paracasei	VCQBS2-1	4	0.5	S	Chinese pickle	Chongqing	SAMN15891426
L. paracasei	VCQYY1-8	4	0.5	S	Chinese pickle	Chongqing	SAMN15891427
L. paracasei	FJSCZD2L3	4	0.5	S	Human feces	Jiangsu	SAMN15891472
L. paracasei	FBJCY3L2	4	0.5	S	Human feces	Beijing	SAMN15891434
L. paracasei	FBJCY4L7	4	0.5	S	Human feces	Beijing	SAMN15891435
L. paracasei	FBJHD18L1	4	0.5	S	Human feces	Beijing	SAMN15891437
L. paracasei	FBJHD3L8	4	0.5	S	Human feces	Beijing	SAMN15891436
L. paracasei	FBJHD50L1	4	0.5	S	Human feces	Beijing	SAMN15891438
L. paracasei	FBJHD53L3	4	0.5	S	Human feces	Beijing	SAMN15891439
L. paracasei	FBJSY36L3	4	0.5	S	Human feces	Beijing	SAMN15891442
L. paracasei	FBJHSY63L1	4	0.5	S	Human feces	Beijing	SAMN15891443
L. paracasei	FBJSY66L5	4	0.5	S	Human feces	Beijing	SAMN15891444
L. paracasei	FHNFQ20L8	4	0.5	S	Human feces	Henan	SAMN15891464
L. paracasei	FJSSZ3L1	4	0.5	S	Human feces	Jiangsu	SAMN15891473
L. paracasei	FNMGWLCB1L2	4	0.5	S	Human feces	Inner Mongolia	SAMN15891476
L. paracasei	FQHXN79L1	4	0.5	S	Human feces	Qinghai	SAMN15891483
L. paracasei	FQHXN83L2	4	0.5	S	Human feces	Qinghai	SAMN15891484
L. paracasei	FQHXN85L3	4	0.5	S	Human feces	Qinghai	SAMN15891486
L. paracasei	FSDHZ5L7	4	0.5	S	Human feces	Shandong	SAMN15891494
L. paracasei	FSDHZ8L2	4	0.5	S	Human feces	Shandong	SAMN15891495
L. paracasei	FSDLZ33L5	4	0.5	S	Human feces	Shandong	SAMN15891497
L. paracasei	FSDLZ34M3	4	0.5	S	Human feces	Shandong	SAMN15891498
L. paracasei	FSDLZ36M1	4	0.5	S	Human feces	Shandong	SAMN15891500
L. paracasei	FSDWF1-2	4	0.5	S	Human feces	Shandong	SAMN15891501
L. paracasei	FXJCJ22M1	4	0.5	S	Human feces	Xinjiang	SAMN15891504
L. paracasei	FXJKS17M1	4	0.5	S	Human feces	Xinjiang	SAMN15891505
L. paracasei	HN13-1	4	0.5	S	Human feces	Hainan	SAMN15891519
L. paracasei	M207F01L6-1-1	4	0.5	S	Human feces	Xizang	SAMN15891522
L. paracasei	NT15-7	4	0.5	S	Human feces	Jiangsu	SAMN15891526
L. paracasei	NT52-1	4	0.5	S	Human feces	Jiangsu	SAMN15891523
L. paracasei	NT72-1	4	0.5	S	Human feces	Jiangsu	SAMN15891524
L. paracasei	NT75-2	4	0.5	S	Human feces	Jiangsu	SAMN15891525
L. paracasei	PS5-4	4	0.5	S	Chinese pickle	-	SAMN15891528
L. paracasei	RS29-1	4	0.5	S	Chinese pickle	Sichuan	SAMN15891530
L. paracasei	RS53-10	4	0.5	S	Chinese pickle	Sichuan	SAMN15891531
L. paracasei	FQHXN106L4	4	0.5	S	Human feces	Qinghai	SAMN15891491
L. paracasei	PCQDXC1-3	4	0.5	S	Chinese pickle	Chongqing	SAMN15891527
L. paracasei	FCQNA38L1	4	0.5	S	Human feces	Chongqing	SAMN15891453
L. paracasei	FCQNA9L2	4	0.5	S	Human feces	Chongqing	SAMN15891447
L. paracasei	FNMHLBE11L4	4	0.5	S	Human feces	Inner Mongolia	SAMN15891478

L. paracasei	FNMHLBE10L1	4	0.5	S	Human feces	Inner Mongolia	SAMN15891477
L. paracasei	FZJHZ24L8	4	0.5	S	Human feces	Zhejiang	SAMN15891512
L. paracasei	FQHXN90L4	4	0.5	S	Human feces	Qinghai	SAMN15891488
L. paracasei	VCQJJ2-96L4	4	0.5	S	Chinese pickle	Chongqing	SAMN15891538
L. rhamnosus	FXJWS19L2	8	64	R	Human feces	Xinjiang	SAMN12429729
L. rhamnosus	FZJHZ4L6	8	8	S	Human feces	Zhejiang	SAMN12429737
L. rhamnosus	FQHXN3M6	8	8	S	Human feces	Qinghai	SAMN12429713
L. rhamnosus	FSHMX1-2	8	8	S	Human feces	Shanghai	SAMN12429715
L. rhamnosus	FJSYC1-5	8	4	S	Human feces	Jiangsu	SAMN12429706
L. rhamnosus	FBJCY2L1	8	2	S	Human feces	Beijing	SAMN12429679
L. rhamnosus	FJSNJ1-1-M2	8	2	S	Human feces	Jiangsu	SAMN12429708
L. rhamnosus	FJSSZ2L1	8	2	S	Human feces	Jiangsu	SAMN12429709
L. rhamnosus	FHeNJZ4L2	8	2	S	Human feces	Henan	SAMN12429688
L. rhamnosus	FHeNJZ8L1	8	2	S	Human feces	Henan	SAMN12429690
L. rhamnosus	FJSZJ2-1	8	2	S	Human feces	Jiangsu	SAMN12429710
L. rhamnosus	FAHWH2L1	8	2	S	Human feces	Anhui	SAMN12429674
L. rhamnosus	FZJHZ14L3	8	2	S	Human feces	Zhejiang	SAMN12429739
L. rhamnosus	FJSWX9L1	8	2	S	Human feces	Jiangsu	SAMN12429701
L. rhamnosus	FJSWX2L6	8	2	S	Human feces	Jiangsu	SAMN12429699
L. rhamnosus	FZJHZD11L1	8	2	S	Human feces	Zhejiang	SAMN12429738
L. rhamnosus	FAHWHD30L7	8	2	S	Human feces	Anhui	SAMN12429676
L. rhamnosus	FWXBH7-3	8	2	S	Human feces	Jiangsu	SAMN12429705
L. rhamnosus	FNMGHLBE18-L5	8	2	S	Human feces	Inner Mongolia	SAMN12429697
L. rhamnosus	FXJWS13L6	8	2	S	Human feces	Xinjiang	SAMN12429728
L. rhamnosus	FXJWS25L4	8	2	S	Human feces	Xinjiang	SAMN12429730
L. rhamnosus	FBJCY3L1	8	1	S	Human feces	Beijing	SAMN12429680
L. rhamnosus	FJSWX24-1	8	1	S	Human feces	Jiangsu	SAMN12429703
L. rhamnosus	FJSWX3-L2	8	1	S	Human feces	Jiangsu	SAMN12429700
L. rhamnosus	FTJDG11-L1	8	1	S	Human feces	Tianjin	SAMN12429722
L. rhamnosus	FH28-1	8	1	S	Human feces	-	SAMN12429694
L. rhamnosus	FJSWX22-4	8	1	S	Human feces	Jiangsu	SAMN12429702
L. rhamnosus	FAHWH35L1	8	1	S	Human feces	Anhui	SAMN12429677
L. rhamnosus	FAHWH38L5	8	1	S	Human feces	Anhui	SAMN12429678
L. rhamnosus	FQHXN4M2	8	1	S	Human feces	Qinghai	SAMN12429714
L. rhamnosus	FZJJH6L2	8	1	S	Human feces	Zhejiang	SAMN12429740
L. rhamnosus	FXJWS3-M4	8	1	S	Human feces	Xinjiang	SAMN12429725
L. rhamnosus	FXJWS2-M1	8	1	S	Human feces	Xinjiang	SAMN12429724
L. rhamnosus	FZJTZ46L6	8	1	S	Human feces	Zhejiang	SAMN12429741
L. rhamnosus	FFJLY7L1	8	1	S	Human feces	Fujian	SAMN12429686
L. rhamnosus	FNMGHLBE6-L3	8	1	S	Human feces	Inner Mongolia	SAMN12429696
L. rhamnosus	FBJSY60L1	8	1	S	Human feces	Beijing	SAMN12429683
L. rhamnosus	FJXSRPYH4L2	8	1	S	Human feces	Jiangxi	SAMN12429711
L. rhamnosus	FSHMX3-1	8	1	S	Human feces	Shanghai	SAMN12429716
L. rhamnosus	FPAL5	8	1	S	Human feces	-	SAMN12429718
L. rhamnosus	FXJWS12L6	8	1	S	Human feces	Xinjiang	SAMN12429727
L. rhamnosus	FXJWS38L2	8	1	S	Human feces	Xinjiang	SAMN12429733

L. rhamnosus	FBJSY7L3	8	0.5	S	Human feces	Beijing	SAMN12429681
L. rhamnosus	FAHWH26-L1	8	0.5	S	Human feces	Anhui	SAMN12429672
L. rhamnosus	FAHWH30-L11	8	0.5	S	Human feces	Anhui	SAMN12429673
L. rhamnosus	FBJSY31L2	8	0.5	S	Human feces	Beijing	SAMN12429682
L. rhamnosus	FFJND15L1	8	0.5	S	Human feces	Fujian	SAMN12429685
L. rhamnosus	FHeNJZ7L1	8	0.5	S	Human feces	Henan	SAMN12429689
L. rhamnosus	FJSYC4-1	8	0.5	S	Human feces	Jiangsu	SAMN12429707
L. rhamnosus	FNMGEL5-1	8	0.5	S	Human feces	Inner Mongolia	SAMN12429695
L. rhamnosus	FJSWX1L3	8	0.5	S	Human feces	Jiangsu	SAMN12429698
L. rhamnosus	FXJSW6-1	8	0.5	S	Human feces	Xinjiang	SAMN12429735
L. rhamnosus	FXJSW24-M2	8	0.5	S	Human feces	Xinjiang	SAMN12429736
L. rhamnosus	FXJWS10-M1	8	0.5	S	Human feces	Xinjiang	SAMN12429726
L. rhamnosus	FGSZY12L6	8	0.5	S	Human feces	Gansu	SAMN12429687
L. rhamnosus	FXJWS44-L2	8	0.5	S	Human feces	Xinjiang	SAMN12429734
L. rhamnosus	FBJSY66L1	8	0.5	S	Human feces	Beijing	SAMN12429684
L. rhamnosus	FSCYA1-L2	8	0.5	S	Human feces	Sichuan	SAMN12429723
L. rhamnosus	FSDLZ7-M12	8	0.5	S	Human feces	Shandong	SAMN12429717
L. rhamnosus	FXJWS37M4	8	0.5	S	Human feces	Xinjiang	SAMN12429732
L. rhamnosus	FJSWX28L2	8	0.5	S	Human feces	Jiangsu	SAMN12429704
L. rhamnosus	FHNFQ4L1	8	0.5	S	Human feces	Henan	SAMN12429692
L. rhamnosus	FHNFQ14L7	8	0.5	S	Human feces	Henan	SAMN12429693
L. rhamnosus	FHNFQ3L5	8	0.5	S	Human feces	Henan	SAMN12429691
L. rhamnosus	FTJDG10-L2	8	0.5	S	Human feces	Tianjin	SAMN12429721
L. rhamnosus	FTJDG4-G3	8	0.5	S	Human feces	Tianjin	SAMN12429719
L. rhamnosus	FTJDG9-L1	8	0.5	S	Human feces	Tianjin	SAMN12429720
L. rhamnosus	FNXYCHL9M3	8	0.25	S	Human feces	Ningxia	SAMN12429712
L. reuteri	FYNLJ86L6	32	>128	R	Dog feces	Yunnan	SAMN15891614
L. reuteri	FSCPS76L4	32	>128	R	Pig feces	Sichuan	SAMN15891589
L. reuteri	FYNLJ87L1	32	>128	R	Pig feces	Yunnan	SAMN15891615
L. reuteri	FYNLJ109L1	32	>128	R	Pig feces	Yunnan	SAMN15891616
L. reuteri	FHNXY12L1	32	>128	R	Chicken feces	Henan	SAMN15891578
L. reuteri	FXJWS25L9	32	>128	R	Human feces	Xinjiang	SAMN15891606
L. reuteri	FYNLJ84L1	32	>128	R	Goose feces	Yunnan	SAMN15891613
L. reuteri	138_1_da	32	>128	R	Human feces	Guangxi	SAMN15891555
L. reuteri	QAHBZ5L1	32	>128	R	Goose feces	Anhui	SAMN15891621
L. reuteri	DYNDL42M2	32	>128	R	Fermented rice milk	Yunnan	SAMN15891566
L. reuteri	FYNDL1_3	32	>128	R	Cow feces	Yunnan	SAMN15891607
L. reuteri	FHNXY64M3	32	>128	R	Human feces	Henan	SAMN21165690
L. reuteri	FJSXYWG3L1	32	>128	R	Cattle feces	Jiangsu	SAMN15891582
L. reuteri	FHNXY71L8	32	>128	R	Chicken feces	Henan	SAMN15891580
L. reuteri	FYNLJ83L8	32	>128	R	Pig feces	Yunnan	SAMN15891612
L. reuteri	FSH14M1	32	>128	R	Human feces	Shanghai	SAMN15891599
L. reuteri	FSCPS79L4	32	>128	R	Dog feces	Sichuan	SAMN15891590
L. reuteri	FXJCJ4M2	32	128	R	Human feces	Xinjiang	SAMN15891604
L. reuteri	FAHBZ3L1	32	128	R	Human feces	Anhui	SAMN15891570
L. reuteri	FNXYCHL81L1	32	64	R	Pigeon feces	Ningxia	SAMN15891585
L. reuteri	FJLHD58L2	32	64	R	Chicken feces	Jilin	SAMN15891581

L. reuteri	FWXBH12M3	32	64	R	Human feces	Jiangsu	SAMN15891603
L. reuteri	DYNDL22M62	32	16	S	Fermented rice milk	Yunnan	SAMN15891565
L. reuteri	FNXYCHL79L1	32	16	S	Cat feces	Ningxia	SAMN15891584
L. reuteri	FGSYC2L3	32	16	S	Human feces	Gansu	SAMN15891575
L. reuteri	FSDLZ13M1	32	8	S	Human feces	Shandong	SAMN15891593
L. reuteri	FYNDL2_1	32	8	S	Human feces	Yunnan	SAMN15891608
L. reuteri	FQHXN127L1	32	8	S	Cattle feces	Qinghai	SAMN15891586
L. reuteri	FHNXY67L4	32	8	S	Chicken feces	Henan	SAMN15891579
L. reuteri	FCQHC8L6	32	8	S	Human feces	Chongqing	SAMN15891571
L. reuteri	FYNDL5_6	32	8	S	Cow feces	Yunnan	SAMN15891609
L. reuteri	DYNDL216	32	8	S	Fermented rice milk	Yunnan	SAMN15891569
L. reuteri	FSDLZ12M1	32	4	S	Human feces	Shandong	SAMN15891592
L. reuteri	FSDLZ8M22	32	4	S	Human feces	Shandong	SAMN15891591
L. reuteri	FSDLZ14M21	32	4	S	Human feces	Shandong	SAMN15891595
L. reuteri	82	32	4	S	Human feces	Jiangsu	SAMN15891552
L. reuteri	72	32	4	S	Human feces	Jiangsu	SAMN15891551
L. reuteri	FSCPS25M1	32	4	S	Human feces	Sichuan	SAMN15891588
L. reuteri	FNMHLBE36L2	32	4	S	Horse feces	Inner Mongolia	SAMN15891583
L. reuteri	51	32	2	S	Human feces	Jiangsu	SAMN15891550
L. reuteri	92	32	2	S	Human feces	Jiangsu	SAMN15891553
L. reuteri	95	32	2	S	Human feces	Jiangsu	SAMN15891554
L. reuteri	311	32	2	S	Rat	Jiangsu	SAMN15891557
L. reuteri	325	32	2	S	Rat feces	Jiangsu	SAMN15891558
L. reuteri	DYNDL11M59	32	2	S	Fermented rice milk	Yunnan	SAMN15891564
L. reuteri	339	32	1	S	Rat feces	Jiangsu	SAMN15891559
L. reuteri	3532	32	1	S	Rat feces	Jiangsu	SAMN15891560
L. plantarum	DHLJZD26L1	32	>128	R	Chinese fermented sauce	Heilongjiang	SAMN15891319
L. plantarum	DHLJZD13L6	32	>128	R	Chinese fermented sauce	Heilongjiang	SAMN15891310
L. plantarum	DHLJZD21L1	32	>128	R	Chinese fermented sauce	Heilongjiang	SAMN15891315
L. plantarum	DHLJZD19L5	32	>128	R	Chinese fermented sauce	Heilongjiang	SAMN15891313
L. plantarum	FHNMY24M8	32	>128	R	Human feces	Henan	SAMN15891347
L. plantarum	VHuNHHMY2L1	32	>128	R	Chinese pickle	Hunan	SAMN15891402
L. plantarum	FSCDJY76L1	32	>128	R	Human feces	Sichuan	SAMN15891366
L. plantarum	FZJTZ19M1	32	128	R	Human feces	Zhejiang	SAMN15891385
L. plantarum	FZJTZ20M1	32	128	R	Human feces	Zhejiang	SAMN15891386
L. plantarum	FZJTZ29M8	32	128	R	Human feces	Zhejiang	SAMN15891387
L. plantarum	FZJTZ31M7	32	128	R	Human feces	Zhejiang	SAMN15891388

L. plantarum	FGDLZ8M3	32	128	R	Human feces	Guangdong	SAMN15891337
L. plantarum	FZJTZ16M7	32	128	R	Human feces	Zhejiang	SAMN15891384
L. plantarum	FSCDJY92L1	32	64	R	Human feces	Sichuan	SAMN15891367
L. plantarum	FCQNA23M1	32	64	R	Human feces	Chongqing	SAMN15891323
L. plantarum	FSCPS6L4	32	64	R	Human feces	Sichuan	SAMN15891369
L. plantarum	FSCPS8L3	32	64	R	Human feces	Sichuan	SAMN15891370
L. plantarum	VCQKX1M2	32	32	S	Chinese pickle	Chongqing	SAMN15891395
L. plantarum	VJLHD12L4	32	32	S	Chinese pickle	Jilin	SAMN15891414
L. plantarum	FSCPS35L5	32	32	S	Human feces	Sichuan	SAMN15891371
L. plantarum	VCQLP6M2	32	32	S	Chinese pickle	Chongqing	SAMN15891396
L. plantarum	FGDLZ10M8	32	32	S	Human feces	Guangdong	SAMN15891339
L. plantarum	FGDLZ9M2	32	32	S	Human feces	Guangdong	SAMN15891338
L. plantarum	VHuNHHMY3L2	32	32	S	Chinese pickle	Hunan	SAMN15891403
L. plantarum	VJXSRYG2L1	32	32	S	Chinese pickle	Jiangxi	SAMN15891419
L. plantarum	FCQNA27M4	32	32	S	Human feces	Chongqing	SAMN15891324
L. plantarum	FCQNA28M4	32	32	S	Human feces	Chongqing	SAMN15891325
L. plantarum	FCQNA29M3	32	32	S	Human feces	Chongqing	SAMN15891326
L. plantarum	FCQNA30M6	32	32	S	Human feces	Chongqing	SAMN15891327
L. plantarum	FCQNA31M2	32	32	S	Human feces	Chongqing	SAMN15891328
L. plantarum	FCQNA32M4	32	32	S	Human feces	Chongqing	SAMN15891329
L. plantarum	FCQNA34M6	32	32	S	Human feces	Chongqing	SAMN15891330
L. plantarum	FCQNA35M1	32	32	S	Human feces	Chongqing	SAMN15891331
L. plantarum	VCQDDK5M2	32	32	S	Chinese pickle	Chongqing	SAMN15891393
L. plantarum	FJLHD20M4	32	32	S	Human feces	Jilin	SAMN15891353
L. plantarum	FJLHD9M2	32	32	S	Human feces	Jilin	SAMN15891351
L. plantarum	FJSCZD4L4	32	32	S	Human feces	Jiangsu	SAMN15891358
L. plantarum	FJSCZD5L9	32	32	S	Human feces	Jiangsu	SAMN15891359
L. plantarum	FXJCJ22M3	32	32	S	Human feces	Xinjiang	SAMN15891372
L. plantarum	FXJCJ25M3	32	32	S	Human feces	Xinjiang	SAMN15891373
L. plantarum	FXJCJ26M6	32	32	S	Human feces	Xinjiang	SAMN15891374
L. plantarum	FXJKS16M1	32	32	S	Human feces	Xinjiang	SAMN15891375
L. plantarum	FXJKS33M3	32	32	S	Human feces	Xinjiang	SAMN15891378
L. plantarum	FXJSW29M6	32	32	S	Human feces	Xinjiang	SAMN15891379
L. plantarum	FXJWS11M2	32	32	S	Human feces	Xinjiang	SAMN15891382
L. plantarum	FXJWS1M3	32	32	S	Human feces	Xinjiang	SAMN15891380
L. plantarum	FXJWS5M4	32	32	S	Human feces	Xinjiang	SAMN15891381
L. plantarum	FCQHC25L4	32	32	S	Human feces	Chongqing	SAMN15891322
L. plantarum	FFJLY44L1	32	16	S	Human feces	Fujian	SAMN15891332
L. plantarum	FCQHC24L1	32	16	S	Human feces	Chongqing	SAMN15891321
L. plantarum	VCQYB1M3	32	16	S	Chinese pickle	Sichuan	SAMN15891398
L. plantarum	DHLJZD23L2	32	16	S	Chinese fermented sauce	Heilongjiang	SAMN15891316

L. plantarum	DHLJZD20L2	32	16	S	Chinese fermented sauce	Heilongjiang	SAMN15891314
L. plantarum	DHLJZD4L1	32	16	S	Chinese fermented sauce	Heilongjiang	SAMN15891309
L. plantarum	DHLJZD16L2	32	16	S	Chinese fermented sauce	Heilongjiang	SAMN15891311
L. plantarum	DHLJZD18L5	32	16	S	Chinese fermented sauce	Heilongjiang	SAMN15891312
L. plantarum	DHLJZD29L2	32	16	S	Chinese fermented sauce	Heilongjiang	SAMN15891320
L. plantarum	DHLJZD24L1	32	16	S	Chinese fermented sauce	Heilongjiang	SAMN15891317
L. plantarum	VJLHD16L1	32	16	S	Chinese pickle	Jilin	SAMN15891416
L. plantarum	VJLHD18L1	32	16	S	Chinese pickle	Jilin	SAMN15891417
L. plantarum	VCQYD1M3	32	16	S	Chinese pickle	Chongqing	SAMN15891400
L. plantarum	VCQWS1M2	32	16	S	Chinese pickle	Chongqing	SAMN15891397
L. plantarum	VCQYB1M3	32	16	S	Chinese pickle	Chongqing	SAMN15891398
L. plantarum	FJLHD37M1	32	16	S	Human feces	Jilin	SAMN15891354
L. plantarum	FJLHD46M1	32	16	S	Human feces	Jilin	SAMN15891355
L. plantarum	FJLHD48M1	32	16	S	Human feces	Jilin	SAMN15891356
L. plantarum	FJLHD4M3	32	16	S	Human feces	Jilin	SAMN15891350
L. plantarum	FJLHD57M1	32	16	S	Human feces	Jilin	SAMN15891357
L. plantarum	FXJKS18M4	32	16	S	Human feces	Xinjiang	SAMN15891376
L. plantarum	FXJKS21M3	32	16	S	Human feces	Xinjiang	SAMN15891377
L. plantarum	FZJTZ60M3	32	16	S	Human feces	Zhejiang	SAMN15891389
L. plantarum	VJLHD14L1	32	16	S	Chinese pickle	Jilin	SAMN15891415
L. plantarum	VJLHD5L1	32	16	S	Chinese pickle	Jilin	SAMN15891409
L. plantarum	VJLHD6L1	32	16	S	Chinese pickle	Jilin	SAMN15891410
L. plantarum	VJLHD7L1	32	16	S	Chinese pickle	Jilin	SAMN15891411
L. plantarum	VJLHD9L1	32	16	S	Chinese pickle	Jilin	SAMN15891412
L. plantarum	VJLHD11L1	32	16	S	Chinese pickle	Jilin	SAMN15891413
L. plantarum	VSCDJY5L5	32	16	S	Chinese pickle	Sichuan	SAMN15891420

L. plantarum	VSCDJY12L1	32	16	S	Chinese pickle	Sichuan	SAMN15891421
L. plantarum	FGSYC22_5_L2	32	16	S	Human feces	Gansu	SAMN15891340
L. plantarum	FHuNHHMY71L1	32	16	S	Human feces	Hunan	SAMN15891348
L. plantarum	FSCDJY69L1	32	16	S	Human feces	Sichuan	SAMN15891364
L. plantarum	FSCDJY93L1	32	16	S	Human feces	Sichuan	SAMN15891368
L. plantarum	VCQDJ1M5	32	8	S	Chinese pickle	Chongqing	SAMN15891394
L. plantarum	DHLJZD25L1	32	8	S	Chinese fermented sauce	Heilongjiang	SAMN15891318
L. plantarum	VCQYC1M1	32	8	S	Chinese pickle	Chongqing	SAMN15891399
L. plantarum	FFJNDD7M7	32	8	S	Human feces	Fujian	SAMN15891333
L. plantarum	FGDLZ1M5	32	8	S	Human feces	Guangdong	SAMN15891334
L. plantarum	FHuNHHMY13M5	32	8	S	Human feces	Henan	SAMN15891345
L. plantarum	FJSWX14L1	32	8	S	Human feces	Jiangsu	SAMN15891361
L. plantarum	FZJTZ61M2	32	8	S	Human feces	Zhejiang	SAMN15891390
L. plantarum	VJLHD4L1	32	8	S	Chinese pickle	Jilin	SAMN15891408
L. plantarum	FSCDJY73L1	32	8	S	Human feces	Sichuan	SAMN15891365
L. plantarum	VCQBB3_125_L8	32	8	S	Chinese pickle	Chongqing	SAMN15891392
L. plantarum	FHuNHHMY22M4	32	4	S	Human feces	Henan	SAMN15891346
L. plantarum	FJLHD2M9	32	2	S	Human feces	Jilin	SAMN15891349
L. plantarum	FGDLZ6M2	32	2	S	Human feces	Guangdong	SAMN15891336
L. plantarum	FHuNHHMY6M5	32	2	S	Human feces	Henan	SAMN15891344
L. plantarum	FZJTZ63M2	32	2	S	Human feces	Zhejiang	SAMN15891391
L. gasseri	FHNXY9_L1	4	16	R	Human feces	Henan	SAMN15891224
L. gasseri	FJXPY24_L2	4	16	R	Human feces	Jiangxi	SAMN15891251
L. gasseri	FJSWX10_4	4	16	R	Human feces	Jiangsu	SAMN15891265
L. gasseri	FJXPY6_L1	4	16	R	Human feces	Jiangxi	SAMN15891249
L. gasseri	AHWH7	4	8	R	Human feces	Anhui	SAMN15891181
L. gasseri	FBJHD4_7	4	8	R	Human feces	Beijing	SAMN15891182
L. paragasseri	FAHBZ9L4	4	8	R	Human feces	Anhui	SAMN21165672
L. gasseri	FFJNDD6_M1	4	8	R	Human feces	Fujian	SAMN15891243
L. gasseri	FGSYC2_L2	4	8	R	Human feces	Gansu	SAMN15891184
L. gasseri	FGSZY12_L1	4	8	R	Human feces	Gansu	SAMN15891197
L. gasseri	FHNFQ25_L3	4	8	R	Human feces	Henan	SAMN15891212
L. gasseri	FHNFQ34_L1	4	8	R	Human feces	Henan	SAMN15891215
L. gasseri	FJSWX6_L7	4	8	R	Human feces	Jiangsu	SAMN15891247
L. paragasseri	FJXPY47L6	4	8	R	Human feces	Jiangxi	SAMN21165685
L. gasseri	FNMGHLBE17_L3	4	8	R	Human feces	Inner Mongolia	SAMN15891256
L. paragasseri	FSCDJY57L4	4	8	R	Human feces	Sichuan	SAMN21165687
L. gasseri	FSCDJY7L1	4	8	R	Human feces	Sichuan	SAMN21165658
L. paragasseri	FSDZB4L1	4	8	R	Human feces	Shandong	SAMN21165688
L. paragasseri	FSDZB5L1	4	8	R	Human feces	Shandong	SAMN21165689

L. gasseri	JSSZ11	4	8	R	Human feces	Jiangsu	SAMN15891263
L. gasseri	JSWX21	4	8	R	Human feces	Jiangsu	SAMN15891266
L. gasseri	JSWX33	4	8	R	Human feces	Jiangsu	SAMN15891267
L. gasseri	QJSWX195M4	4	8	R	Human vagina	Jiangsu	SAMN15891272
L. gasseri	QJSWX309M31	4	8	R	Human vagina	Jiangsu	SAMN15891274
L. gasseri	FGSYC9	4	8	R	Human feces	Gansu	SAMN15891187
L. gasseri	FGSYC19	4	8	R	Human feces	Gansu	SAMN15891191
L. gasseri	FGSZY27	4	8	R	Human feces	Gansu	SAMN15891198
L. gasseri	FGSZY30	4	8	R	Human feces	Gansu	SAMN15891200
L. gasseri	FHNFQ15	4	8	R	Human feces	Henan	SAMN15891209
L. gasseri	SDHZD3	4	8	R	Human feces	Shandong	SAMN15891275
L. gasseri	SDYT1	4	8	R	Human feces	Shandong	SAMN15891276
L. gasseri	FJXPY37	4	8	R	Human feces	Jiangxi	SAMN15891254
L. gasseri	FGSYC18_L5	4	4	S	Human feces	Gansu	SAMN15891190
L. gasseri	FGSYC43_L1	4	4	S	Human feces	Gansu	SAMN15891195
L. gasseri	AHFY1	4	4	S	Human feces	Anhui	SAMN15891180
L. paragasseri	FBJCP2L3	4	4	S	Human feces	Beijing	SAMN21165673
L. gasseri	FGSYC34_L2	4	4	S	Human feces	Gansu	SAMN15891192
L. paragasseri	FGSYC4L2	4	4	S	Human feces	Gansu	SAMN21165676
L. gasseri	FGSYC79_L2	4	4	S	Human feces	Gansu	SAMN15891196
L. paragasseri	FGSZY31L4	4	4	S	Human feces	Gansu	SAMN21165677
L. gasseri	FHeBCZ3_L3	4	4	S	Human feces	Hebei	SAMN15891202
L. paragasseri	FHeNWX1L5	4	4	S	Human feces	Henan	SAMN21165678
L. paragasseri	FHNFQ10L1	4	4	S	Human feces	Henan	SAMN21165680
L. gasseri	FFJND16L4	4	4	S	Human feces	Fujian	SAMN21165655
L. gasseri	FJXPY26_L4	4	4	S	Human feces	Jiangxi	SAMN15891252
L. paragasseri	FNXHC13_L3	4	4	S	Human feces	Ningxia	SAMN21165686
L. gasseri	FJSCZD2L1	4	4	S	Human feces	Jiangsu	SAMN21165656
L. gasseri	M2CF21L1	4	4	S	Human feces	Xizang	SAMN21165659
L. gasseri	QJSWX163M1	4	4	S	Human vagina	Jiangsu	SAMN21165660
L. gasseri	QJSWX166M6	4	4	S	Human vagina	Jiangsu	SAMN21165661
L. gasseri	QJSWX306M13	4	4	S	Human vagina	Jiangsu	SAMN15891273
L. gasseri	FGSZY36	4	4	S	Human feces	Gansu	SAMN15891201
L. gasseri	FHNFQ3	4	4	S	Human feces	Henan	SAMN15891205
L. gasseri	FHNFQ11	4	4	S	Human feces	Henan	SAMN15891207
L. gasseri	FHNFQ14	4	4	S	Human feces	Henan	SAMN15891208
L. gasseri	FHNFQ16	4	4	S	Human feces	Henan	SAMN15891210
L. gasseri	FHNFQ20	4	4	S	Human feces	Henan	SAMN15891211
L. gasseri	FHNFQ28	4	4	S	Human feces	Henan	SAMN15891213
L. gasseri	FHNFQ29	4	4	S	Human feces	Henan	SAMN15891214
L. gasseri	FHNFQ53	4	4	S	Human feces	Henan	SAMN15891217
L. gasseri	FHNFQ57	4	4	S	Human feces	Henan	SAMN15891219
L. gasseri	FHLJDQ3	4	4	S	Human feces	Heilongjiang	SAMN15891204
L. paragasseri	FFJND2L7	4	4	S	Human feces	Fujian	SAMN21165675
L. gasseri	ZJHZD1	4	4	S	Human feces	Zhejiang	SAMN15891277

L. gasseri	FHNXY46_L6	4	4	S	Human feces	Henan	SAMN15891232
L. gasseri	FJFZ1	4	4	S	Human feces	Fujian	SAMN15891240
L. gasseri	FHNXY58_L2	4	4	S	Human feces	Henan	SAMN15891237
L. gasseri	FHNXY29_L1	4	4	S	Human feces	Henan	SAMN15891229
L. gasseri	FHNXY56_L1	4	4	S	Human feces	Henan	SAMN15891236
L. gasseri	FHNXY61_L1	4	4	S	Human feces	Henan	SAMN15891238
L. gasseri	FHNXY54_L2	4	4	S	Human feces	Henan	SAMN15891235
L. gasseri	FHNXY44_L1	4	4	S	Human feces	Henan	SAMN15891231
L. gasseri	FHNXY49_L5	4	4	S	Human feces	Henan	SAMN15891233
L. paragasseri	FJSWX34JL3	4	4	S	Human feces	Jiangsu	SAMN21165683
L. paragasseri	FJXPY27L6	4	4	S	Human feces	Jiangxi	SAMN21165684
L. gasseri	FGSYC8_L2	4	4	S	Human feces	Gansu	SAMN15891186
L. gasseri	FGSYC38_L3	4	2	S	Human feces	Gansu	SAMN15891193
L. gasseri	FGSYC7_L1	4	2	S	Human feces	Gansu	SAMN15891185
L. gasseri	FGSYC10	4	2	S	Human feces	Gansu	SAMN15891188
L. gasseri	FGSYC15	4	2	S	Human feces	Gansu	SAMN15891189
L. gasseri	FGSZY29	4	2	S	Human feces	Gansu	SAMN15891199
L. gasseri	FHNXY28_L4	4	2	S	Human feces	Henan	SAMN15891228
L. gasseri	FHNFQ60_L1	4	2	S	Human feces	Henan	SAMN15891220
L. gasseri	C1A31	4	2	S	Human feces	Henan	SAMN21165654
L. gasseri	FHNXY34_L1	4	2	S	Human feces	Henan	SAMN15891230
L. paragasseri	FCQHC7L6	4	2	S	Human feces	Chongqing	SAMN21165674
L. paragasseri	FHNXY27L2	4	2	S	Human feces	Henan	SAMN21165681
L. gasseri	FHNXY52_L2	4	2	S	Human feces	Henan	SAMN15891234
L. paragasseri	FHeNXX1L6	4	1	S	Human feces	Henan	SAMN21165679
L. paragasseri	FJSCZD12L9	4	1	S	Human feces	Jiangsu	SAMN21165682
L. gasseri	QJSWX174M8	4	1	S	Human vagina	Jiangsu	SAMN21165662
L. gasseri	FJSCZD7_L5	4	1	S	Human feces	Jiangsu	SAMN21165657
L. gasseri	FGSY23_L3	4	0.25	S	Human feces	Gansu	SAMN15891183
L. gasseri	FHNFQ46_L1	4	0.25	S	Human feces	Henan	SAMN15891216
L. gasseri	FHNFQ62_L6	4	0.25	S	Human feces	Henan	SAMN15891221
L. gasseri	FHNFQ63_L6	4	0.25	S	Human feces	Henan	SAMN15891222
L. gasseri	FHNXY12_L2	4	0.25	S	Human feces	Henan	SAMN15891225
L. gasseri	FHNXY18_L2	4	0.25	S	Human feces	Henan	SAMN15891226
L. gasseri	FHNXY26_L3	4	0.25	S	Human feces	Henan	SAMN15891227
L. gasseri	FGSYC41	4	0.25	S	Human feces	Gansu	SAMN15891194
L. johnsonii	FHNXY70M2	4	128	R	Chicken feces	Henan	SAMN15891283
L. johnsonii	FSDQZ12L4	4	64	R	Human feces	Shandong	SAMN21165665
L. johnsonii	QJSWX307M3	4	64	R	Human vagina	Jiangsu	SAMN21165669
L. johnsonii	FSCPS86M4	4	64	R	Human feces	Sichuan	SAMN15891285
L. johnsonii	FJLHD57L1	4	64	R	Human feces	Jilin	SAMN21165664
L. johnsonii	FHBZX13M2	4	64	R	Human feces	Hubei	SAMN15891282
L. johnsonii	VJSWX307M4	4	64	R	Human vagina	Jiangsu	SAMN15891288
L. johnsonii	VJSWX309M14	4	64	R	Human vagina	Jiangsu	SAMN21165670
L. johnsonii	QJSWX125M11	4	64	R	Human vagina	Jiangsu	SAMN21165667

L. johnsonii	VJSWX316M31	4	64	R	Human vagina	Jiangsu	SAMN21165671
L. johnsonii	FAHBZ615	4	64	R	Human feces	Anhui	SAMN15891280
L. johnsonii	FHUBES1M16	4	64	R	Human feces	Hubei	SAMN21165663
L. johnsonii	QGSYC2L2	4	64	R	Chicken feces	Gansu	SAMN15891286
L. johnsonii	JCM1101	4	8	R	JCM	-	SAMN21165666
L. johnsonii	QJSWX160M2	4	8	R	Human vagina	Jiangsu	SAMN15891287
L. johnsonii	QJSWX159M2	4	4	S	Human vagina	Jiangsu	SAMN21165668
L. johnsonii	FJLHD58L1	4	4	S	Human feces	Jilin	SAMN15891284
L. johnsonii	FGSYC6M3	4	2	S	Human feces	Gansu	SAMN15891281
L. crispatus	FHNXY70M14	4	>128	R	Chicken feces	Henan	SAMN12869313
L. crispatus	FHuNAHMY89L4	4	>128	R	Pig feces	Hunan	SAMN15891006
L. crispatus	FSCPS74L3	4	>128	R	Pig feces	Sichuan	SAMN15891009
L. crispatus	FCQJJ9M2	4	>128	R	Chicken feces	Chongqing	SAMN12869312
L. crispatus	FHNXY35M5	4	>128	R	Human feces	Henan	SAMN12869307
L. crispatus	QJSWX198M4	4	>128	R	Human vagina	Jiangsu	SAMN15891012
L. crispatus	QJSWX200M1	4	>128	R	Human vagina	Jiangsu	SAMN15891011
L. crispatus	QAHBZ6L1	4	>128	R	Duck feces	Anhui	SAMN15891010
L. crispatus	FHNFQ29L3	4	>128	R	Human feces	Henan	SAMN12869303
L. crispatus	FHNXY45L1	4	>128	R	Human feces	Henan	SAMN12869308
L. crispatus	FHUBES1M17	4	>128	R	Human feces	Hubei	SAMN21165652
L. crispatus	FHNFQ45L4	4	>128	R	Human feces	Henan	SAMN12869305
L. crispatus	QGSYC6L6	4	128	R	Chicken feces	Gansu	SAMN12869319
L. crispatus	FHNFQ37L2	4	128	R	Human feces	Henan	SAMN12869304
L. crispatus	FHNXY64M2	4	128	R	Chicken feces	Henan	SAMN15891005
L. crispatus	FJLHD56L3	4	64	R	Chicken feces	Jilin	SAMN12869315
L. crispatus	FSCPS86L2	4	64	R	Chicken feces	Sichuan	SAMN12869317
L. crispatus	FHNXY56M7	4	8	R	Human feces	Henan	SAMN15891004
L. crispatus	QJSWX169M3	4	4	S	Human vagina	Jiangsu	SAMN12869332
L. crispatus	JCM2009	4	4	S	JCM	-	SAMN21165653
L. crispatus	FAHXC2L1	4	4	S	Chicken feces	Anhui	SAMN12869311
L. crispatus	FSCDJY13L2	4	4	S	Human feces	Sichuan	SAMN15891008
L. crispatus	FHNFQ56M4	4	4	S	Human feces	Henan	SAMN12869306
L. crispatus	QJSWX150M37	4	4	S	Human vagina	Jiangsu	SAMN12869329
L. crispatus	FGSYC18L1	4	4	S	Human feces	Gansu	SAMN12869300
L. crispatus	QJSWX112M2	4	2	S	Human vagina	Jiangsu	SAMN12869323
L. crispatus	QJSWX174M9	4	2	S	Human vagina	Jiangsu	SAMN12869333
L. crispatus	QJSWX109M4	4	2	S	Human vagina	Jiangsu	SAMN12869321
L. crispatus	QJSWX166M13	4	2	S	Human vagina	Jiangsu	SAMN12869331
L. crispatus	QJSWX110M1	4	1	S	Human	Jiangsu	SAMN12869322

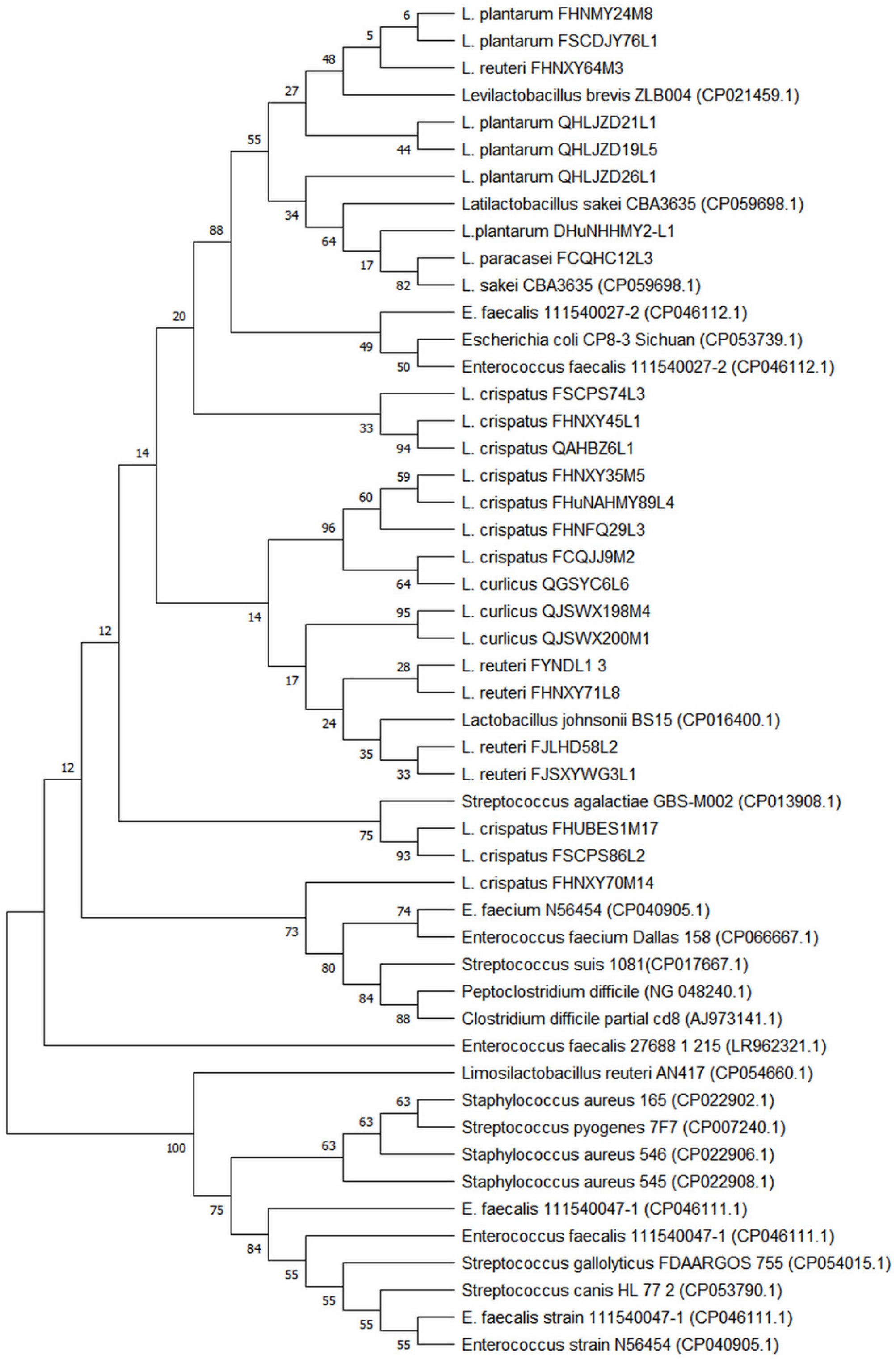

Figure S1. Phylogenetic analysis of tet(M) belonging to 25 strains of lactic acid bacteria identified in this paper together with the same genotype sequences retrieved from NCBI database.

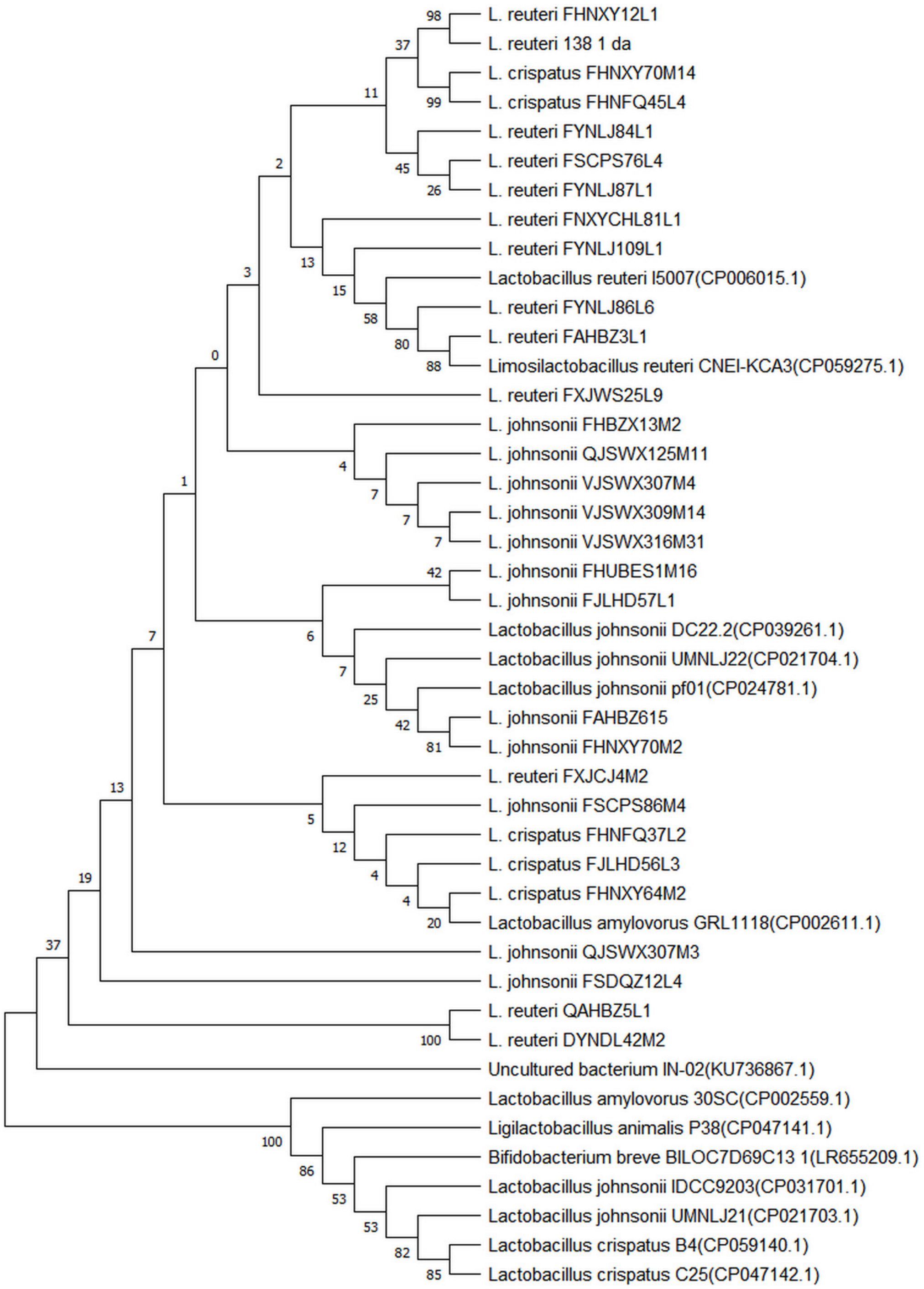

Figure S2. Phylogenetic analysis of tet(W/N/W) belonging to 30 strains of lactic acid bacteria identified in this paper together with the same genotype sequences retrieved from NCBI database.

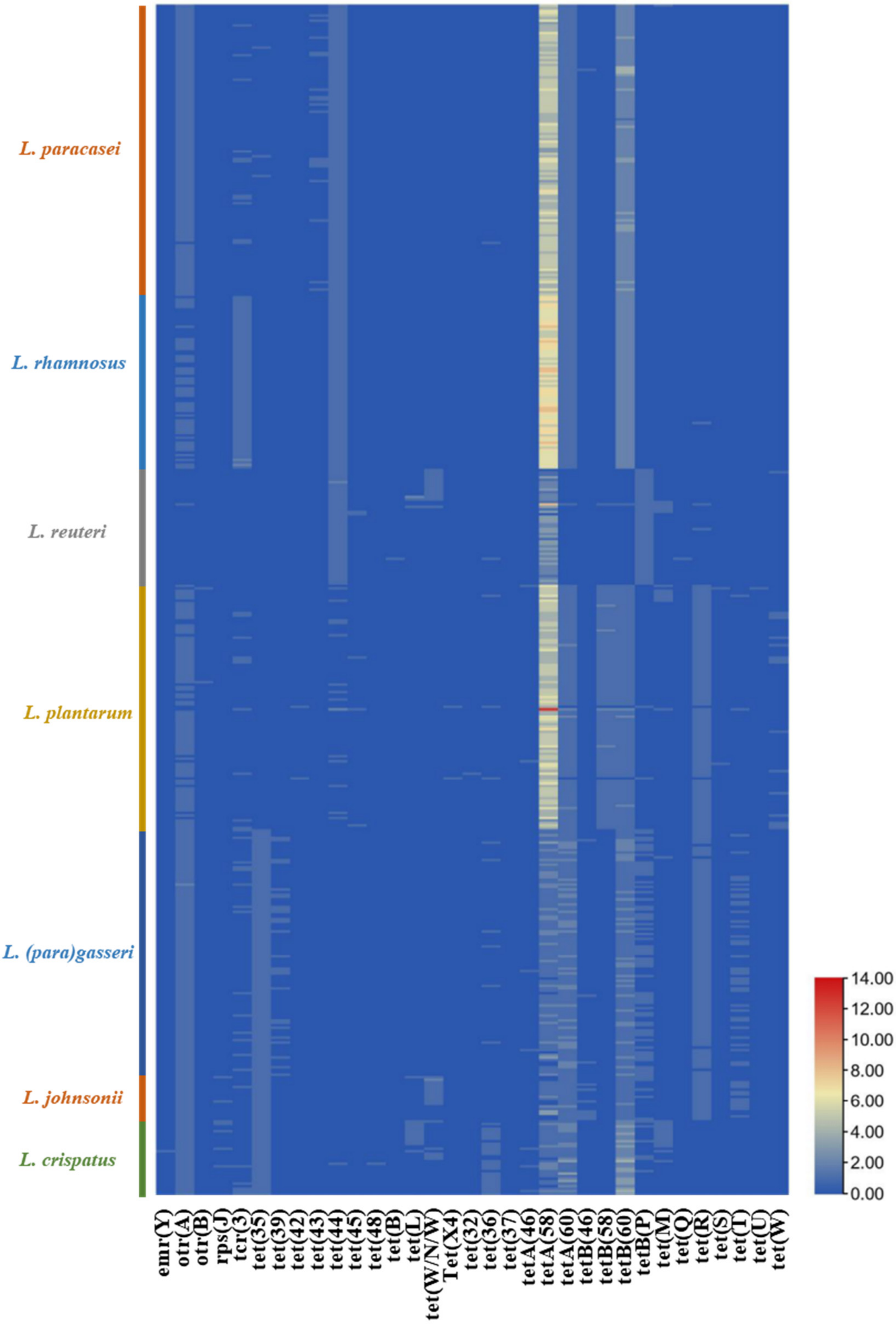

Figure S3. The resistance genes of 478 strains of lactic acid bacteria were detected with an identity greater than 30% as the threshold.
